# Ultra High Strain Rate Nanoindentation Testing

**DOI:** 10.3390/ma10060663

**Published:** 2017-06-17

**Authors:** Pardhasaradhi Sudharshan Phani, Warren Carl Oliver

**Affiliations:** 1 International Advanced Research Centre for Powder Metallurgy and New Materials (ARCI), Balapur PO, Hyderabad, Telangana 500005, India; 2 Nanomechanics Inc., 105 Meco Ln, Oak Ridge, TN 37830, USA; warren.oliver@nanomechanicsinc.com

**Keywords:** high strain rate, nanoindentation, aluminum alloy, dynamics

## Abstract

Strain rate dependence of indentation hardness has been widely used to study time-dependent plasticity. However, the currently available techniques limit the range of strain rates that can be achieved during indentation testing. Recent advances in electronics have enabled nanomechanical measurements with very low noise levels (sub nanometer) at fast time constants (20 µs) and high data acquisition rates (100 KHz). These capabilities open the doors for a wide range of ultra-fast nanomechanical testing, for instance, indentation testing at very high strain rates. With an accurate dynamic model and an instrument with fast time constants, step load tests can be performed which enable access to indentation strain rates approaching ballistic levels (i.e., 4000 1/s). A novel indentation based testing technique involving a combination of step load and constant load and hold tests that enables measurement of strain rate dependence of hardness spanning over seven orders of magnitude in strain rate is presented. A simple analysis is used to calculate the equivalent uniaxial response from indentation data and compared to the conventional uniaxial data for commercial purity aluminum. Excellent agreement is found between the indentation and uniaxial data over several orders of magnitude of strain rate.

## 1. Introduction

Measuring the strain rate dependence of flow stress is of great interest to the materials community and has been a widely-studied research area [1]. The strain rate dependence of flow stress of bulk materials can be routinely measured over a wide range using many conventional techniques like uniaxial compression/tension for lower strain rates and Split-Hopkinson pressure bar for high strain rates [2]. However, these techniques are not readily applicable for small-scale structures or small volumes of materials, which has been a recent area of focus for the materials community. Several groups have used micro/nano impact testing [3,4,5] or dynamic indentation to understand the high strain rate behavior without necessarily using the depth sensing capability and they mostly fall under the microindentation regime [6,7,8,9,10,11]. Techniques based on nanoindentation such as constant strain rate test, strain rate jump test, constant rate of loading test, or a constant load and hold test have been widely used to measure the rate dependence of hardness of small volumes of materials over a range of strain rates [12,13,14,15,16]. These techniques are typically limited to the lower strain rate regimes (<1 1/s). In order to access high strain rates during indentation, a step load test can be performed, wherein, the load is ramped within a few micro seconds [14]. This results in sweeping across a wide range of strain rates especially in the high strain rate regime (>100 1/s) in a single test. This could be a powerful technique to measure the high strain rate response at small scales in a simple, quick, and cost-effective way.

While the step load test theoretically offers a great opportunity to probe the material response at high strain rates, there are several experimental challenges, such as the dynamic contribution of the instrument and the time constants of the measurement signals, which need to be carefully considered in order to make valid measurements. Recent advances in electronics have enabled nanomechanical measurements with very low noise levels (sub nanometer) at fast time constants (20 µs) and high data acquisition rates (100 KHz). These capabilities open the doors for a wide range of ultra-fast nanomechanical testing. In addition to having a measuring instrument with fast response, a comprehensive model for the dynamics of the instrument and measurement electronics is required to make accurate high strain rate measurements. In this work, we present a step load-based indentation high strain rate measurement technique that relies on fast response instrumentation and a comprehensive model for the instrument’s dynamics and electronics. High strain rate tests are performed on annealed commercial purity aluminum alloy (1100 Al) to demonstrate the technique. A simple analysis is used to calculate the uniaxial equivalent response from the indentation results and is compared to the conventional high strain rate tests to assess the accuracy of this technique. The relative contribution of the instrument’s dynamic response and the time constants of the measurement signals to the overall measurement are also discussed to demonstrate the importance of accurate instrument characterization for high strain rate indentation testing.

## 2. Experimental Procedure and Calculations

### 2.1. Measuring Strain Rate Dependence of Hardness

As mentioned earlier, indentation based techniques have been widely used to measure the strain rate dependence of hardness in the lower strain rate regime (<1 1/s). The strain rate during an indentation test is often defined as the ratio of the indenter velocity to the depth of indentation. Accessing higher strain rates requires higher indentation velocities at a given depth, or a lower depth for a given velocity or a combination of both. In order to minimize the contributions from indentation size effect (ISE) [17], it is preferable to access higher strain rates by achieving higher velocities at large depths. This can be accomplished by performing a step load test wherein the force is ramped as fast as the actuator used can physically accomplish the change. This results in sweeping a wide range of strain rates in the high strain rate regime in a single indentation test. The major requirements to perform these tests is a testing system with fast response actuators and sensors and a model for instrument’s dynamics and electronics to accurately factor out the instrument’s contribution from the measured response. These will be described in greater detail in the subsequent subsections.

Step load tests and the conventional constant load and hold (CLH) indentation tests were performed to a static load of 16 mN to cover a wide range of indentation strain rates. In both the cases, the tip is brought in contact with the sample at a slow approach rate of 200 nm/s. The CLH tests were performed by ramping the load to 16 mN at a loading rate of 5 N/s after contact and subsequently maintaining a constant force for 30 s. In the case of step load tests, a step force of 16 mN was input to the force actuator after contact. Note that the step load tests are not impact tests as the tip approaches the sample slowly before contact and the fast loading is only after contact. Unlike the CLH test, due to the fast loading in a step load test, there are significant inertial effects which result in actual load on sample being much higher than the applied step force of 16 mN for a short span. This is immediately followed by a decrease in the load on the sample due to the exhaustion of the dynamic forces, resulting in unloading of the contact even while the actuator applies the 16 mN force. This will be discussed in greater detail in Section 3.1. All the tests were performed on a 10 mm diameter commercial purity aluminum (1100 aluminum) sample which was polished and subsequently annealed at 350 °C for 4 h before testing. Ten repetitive step load and CLH tests were performed to ensure repeatability in the data. A diamond Berkovich tip (Micro Star Technologies, Huntsville, TX, USA) was used for all the tests. The load frame stiffness determination and tip area calibration was done using the results of constant strain rate tests (0.2 1/s) on fused silica. For these tests, the contact stiffness was continuously measured as a function of depth using a phase lock amplifier (Nanomechanics Inc., Oak Ridge, TN, USA) oscillating at 100 Hz frequency and a 2 nm displacement amplitude.

### 2.2. Measuring Instrumentation

In order to perform a step load test, the testing system requires a force actuator that can apply the desired force in a short time interval, which is typically less than a millisecond, and a displacement sensor that can accurately capture the rapid change in the displacement during that time. In addition, a high data acquisition rate is required. In a typical commercially-available nanoindentation system with an electromagnetic actuator, force is controlled by the current to the coil, which is a command signal, and the displacement is measured by a capacitance gage, which is a measured signal. To perform a step load test, a step function in current is sent as a command input to the actuator. Due to the finite time constant of the force signal, the actual force delivered by the actuator is not an instantaneous step function, but an exponential function with a finite rise time. The time constant of a signal is a parameter that characterizes the response of a signal to a unit step input. For first order linear-time invariant systems, it is the time required to reach 63% of its step input value or one third the time required to reach 95% of its step input. Hence, if the time scale of the test is comparable to the time constants of the measurement signals, corrections to the signals are required. In order to minimize these corrections, it is desirable to have measurement time constants much shorter than the time scale of measurement.

The step load tests in the current work were performed using a commercially available nanoindenter, iNano^®^ from Nanomechanics Inc., Oak Ridge, TN, USA. It uses an electromagnetically-actuated InForce50 actuator with a force time constant of 290 µs, a displacement time constant of 20 µs, and data acquisition rate of 100 kHz. Force is the command signal and the displacement is the measured signal. The displacement sensor has sub nanometer noise levels even at a short time constant of 20 µs. This is critical for high strain rate testing as the velocity and acceleration are calculated by taking the first and second derivative of the displacement signal and any noise is amplified, especially by the second derivative.

### 2.3. Model for the Instrument’s Dynamics and Electronics

**Figure 1:**
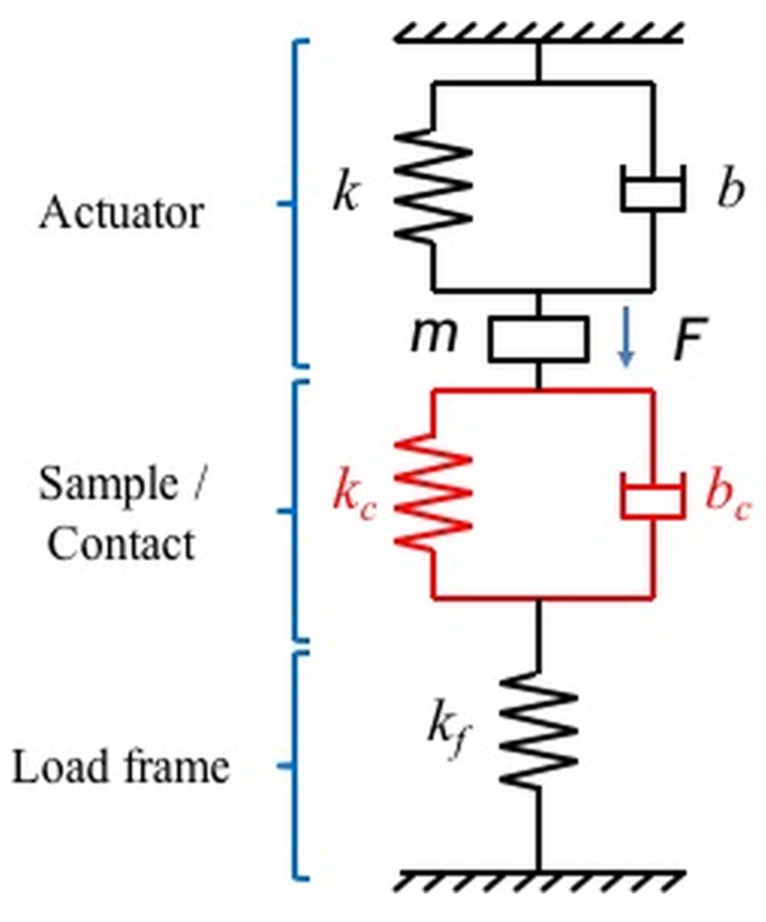
Schematic of the one DOF model for the indentation system showing the various dynamic elements used for the actuator, sample/contact, and the load frame.

At high strain rates, the instrument’s dynamic contribution can dominate the measured response and can lead to inaccuracies in the measurement. In order to account for the instrument’s dynamic contribution, a simple one degree of freedom (one DOF) damped harmonic oscillator model is proposed to model the electromagnetically-actuated indentation system. A schematic of the model is shown in Figure 1. The actuator is modeled as a single mass, spring, and dashpot system where the mass, *m*, is the moving mass of the coil and the indenter shaft, damping coefficient, *b*, is the damping generated due to resistance to the motion of the air in the capacitance gage and the eddy current damping in an electromagnetic actuator and the spring constant, *k*, is the spring constant of the leaf springs that support the indenter shaft. The sample or the contact is modeled as a spring and dashpot, which represents the contact stiffness and damping, respectively. As the moving mass of the sample is very small compared to the mass of the system, it is neglected. The load frame which holds the indentation system is modeled as a spring for simplicity.

The one DOF model shown in Figure 1 is one of the simplest possible dynamic models for an indentation system and given the complexity of most indentation systems, demonstrating that the testing system can be accurately described by this model is critical for high strain rate testing where the instruments contribution can dominate the measured response. Once the simple model for the actuator is validated its dynamic contribution can be simply factored out to accurately determine the response of the sample.

**Figure 2:**
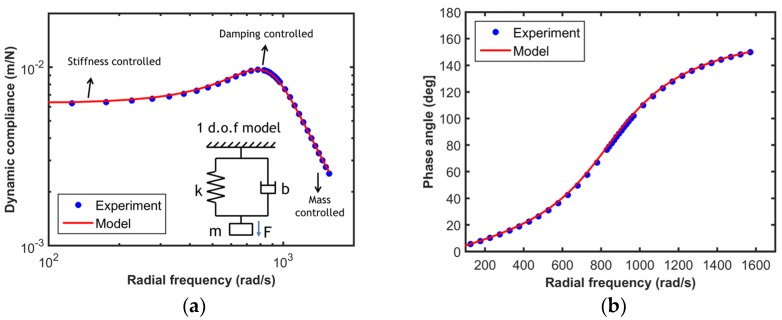
Comparison of experimental data and one DOF model data for (**a**) dynamic compliance, and (**b**) phase angle of the actuator, as a function of radial frequency for a typical InForce50 actuator.

In order to validate the one DOF model for the actuator, a frequency sweep experiment is performed, wherein the actuator is excited dynamically at a fixed sinusoidal force oscillation amplitude over a wide range of frequency in free air (i.e., without a sample), The resultant dynamic displacement amplitude and the phase lag between the force and displacement signals are measured using a phase lock amplifier (PLA). Figure 2a shows the results of a typical frequency sweep experiment for an InForce50 actuator, wherein the measured dynamic compliance of the instrument in free air, which is the ratio of the dynamic displacement amplitude ($h_{o}$) to the dynamic force amplitude ($F_{o}$), is plotted as a function of the excitation frequency (ω). This is commonly referred to as the transfer function of the instrument. The dynamic compliance (*C*) and phase (*ø*) for a one DOF oscillator can be theoretically calculated using the following equations.

(1) $$C=h_{o}/F_{o}\;=\sqrt{\left({\left(k-m\omega^{2}\right)}^{2}+{\left(b\omega \right)}^{2}\right)}$$

(2) $$\phi =\tan^{-1}\left(\frac{b\omega }{k-m\omega^{2}}\right)$$

The experimental data shown in the plot can be fit to a functional form given in Equation (1) to assess the suitability of using the one DOF model for the instrument. The solid red line in the plot shows the one DOF model fit to the experimental data. This plot clearly demonstrates that the actuator can be accurately modeled as a simple one DOF oscillator. The mass (*m*), damping coefficient (*b*), and spring constant (*k*) of the actuator used for the current work are 180.45 mg, 0.106 Ns/m, and 243 N/m, respectively. Figure 2b shows the experimental data and the model prediction for the phase angle between the displacement and force signals, reinforcing the excellent agreement in the results observed from the transfer function plot and also demonstrating the accuracy of the phase angle measurements. While the one DOF model presented here is simple, it can even be used under non-ambient conditions (vacuum or elevated temperature) for electromagnetic actuators as they do not require a medium for damping. This enables high strain rate experiments under vacuum or elevated temperatures.

In addition to the dynamic effects of the instrument, understanding and accounting for the time constants of the measurement signals is important for making accurate high strain rate measurements. As discussed in Section 2.2, time constant correction to the signals is required if the time scale of testing is comparable to the measurement time constant. A simple first order correction can be performed to the signal to account for the finite time constant, by the following equation:

(3) $$y_{corr}=y_{msd}+\left(y_{msd}^{\;˙}\right)/2\tau$$

where $y_{corr}$ is the corrected signal, $y_{msd}\;$is the measured signal, $\dot{y_{msd}}$ is the rate of change of the measured signal, and τ is the time constant.

**Figure 3:**
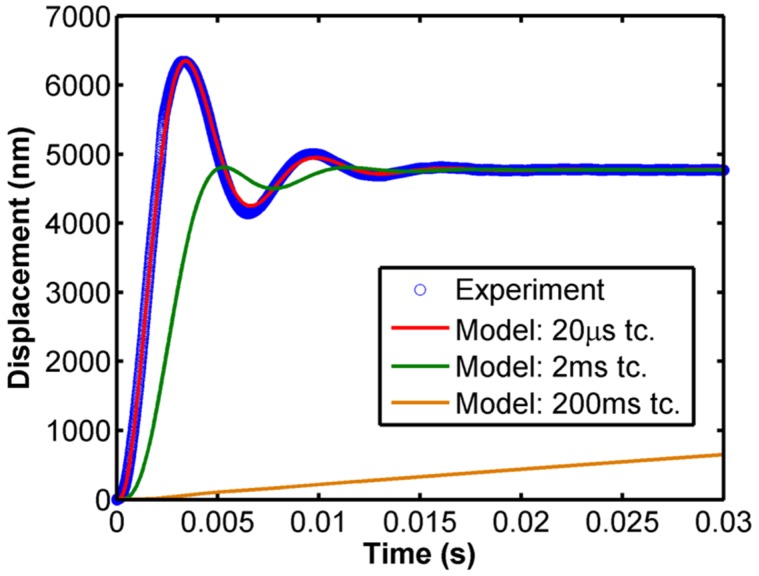
Comparison of experimental data and model predictions at different displacement time constants (tc.) for the step load response of the actuator in free air for a typical InForce50 actuator.

While the transfer function plot demonstrates that the instrument can be modeled as a one DOF oscillator, it does not prove the accuracy of the model parameters *m*, *b*, and *k* when applied in conjunction with the time constants of the force and displacement signals which can have a significant effect during fast testing. In order to verify the accuracy of the time constant correction and dynamic model, a step load test is performed in free air wherein the force command signal is instantaneously stepped to 1 mN and the resultant displacement response is recorded at 100 kHz. Figure 3 shows the comparison of the experimental data for a typical InForce50 actuator and model prediction for a one DOF model at different time constants. There is excellent agreement between the experimental data and model predictions for a 20 µs time constant displacement signal. This demonstrates the validity of the model for the instrument’s dynamics, as well as the electronics and, hence, can be used to accurately factor out the contribution of the instrument to the total measurement which will be discussed in greater detail in Section 2.4. The plot also shows the predicted response for 1 ms and 200 ms time constant displacement signals which show significant deviation from the actual response. Note that the data acquisition rate for all of the curves is 100 kHz and the difference is only in the time constant. While having a high data acquisition rate is important, this plot clearly demonstrates the need for a short time constant to accurately capture dynamic events.

### 2.4. Calculation of the Indentation Strain Rate and Hardness

This section presents the procedure for calculating the indentation strain rate and hardness from the basic measurements, viz., force and displacement. As mentioned earlier, indentation strain rate is the ratio of indenter velocity ($\dot{h}$) to the depth of penetration (*h*). The depth of penetration is calculated by subtracting the displacement of the surface point from the measured displacement. The velocity ($\dot{h}$) and acceleration ($\ddot{h}$) are calculated by taking the analytical first and second derivative, respectively, of the spline fit to the displacement-time response. In order to calculate the load on the sample, the dynamic contribution of the instrument needs to be factored out. This can be done by using the simple one DOF model for the actuator which has been shown to accurately describe the system in Section 2.3. The equation to calculate the load on the sample (*P*) factoring out the dynamic contribution of the instrument, is as follows:

(4) $$P=F-kh-b\dot{h}-m\ddot{h}$$

where *F* is the force output of the actuator and *h* is the depth of penetration into the surface. Note that the load on the sample can be very different from the force output of the actuator depending on the dynamic contributions of the instrument. This difference is quite significant for the step load tests where the inertial term ($m\ddot{h}$) dominates. For the case of the constant load and hold tests the contribution from the damping ($b\dot{h}$) and inertial terms is negligible as expected for static indentation.

Once the load on sample is calculated from Equation (4), the hardness is calculated using the conventional formula of load over the contact area. The contact area is calculated from the tip area function assuming a constant ratio of contact depth to total depth which, for this case, is found to be 0.99 based on the unloading data of the CLH tests.

### 2.5. Estimating Equivalent Uniaxial Response

The experimental procedure and calculations described in the earlier sections enable the determination of hardness as a function of the indentation strain rate. However, in order to compare the data to the conventional measurements, which are based on uniaxial testing, uniaxial equivalent parameters have to be estimated from the indentation data, which is challenging given the complexity of the stress fields during indentation. Recently, Su et al. [18] proposed a simple experimental technique based on the theoretical analysis of Bower et al. [19] to determine uniaxial creep parameters from indentation. The equations used to calculate the uniaxial equivalent strain rate and stress are presented here while the details can be found in Su et al. [18]. The equivalent uniaxial strain rate ($\dot{\varepsilon }$) and stress (σ) for a power-law creeping solid can be calculated from the basic indentation measurements (*h* and *P*) using the following equations:

(5) $$\dot{\varepsilon }=\left(\frac{1}{c\tan\theta }\right)\left(\frac{1}{h}\right)\left(\frac{dh}{dt}\right)$$

(6) $$\sigma =\left(\frac{1}{Fc^{2}}\right)\left[\frac{P}{\pi {\left(h\tan\theta \right)}^{2}}\right]$$

In the above equations, θ is the equivalent half cone angle, which is 70.3° for a Berkovich indenter and *F* and *c* are akin to constraint factor and pile-up/sink-in parameter, respectively. *F* and *c* are a function of the stress exponent and cone angle, and their functional dependence can be obtained from the recent work of Su et al. [18].

## 3. Results and Discussion

### 3.1. Step Load and Constant Load and Hold Tests: Basic Measurements

**Figure 4:**
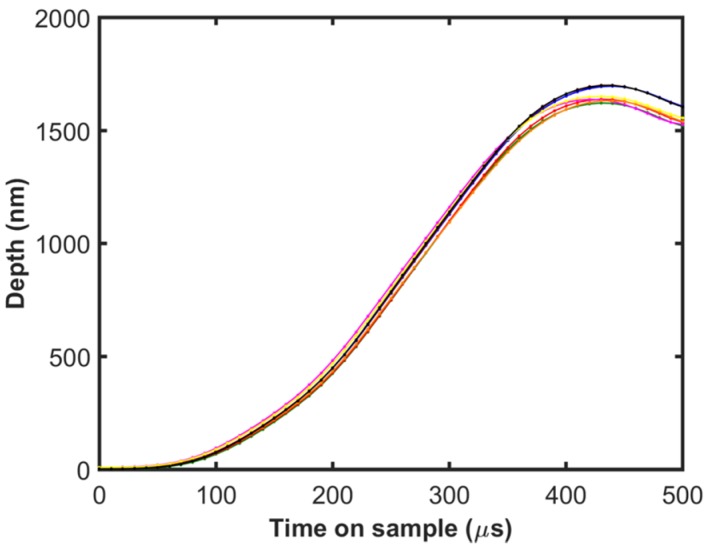
Indentation depth as a function of the time on the sample during the step force input of 16 mN for 10 different tests shown in different colors.

The depth of penetration into the sample as a function of the time on the sample in response to a 16 mN step force input is shown in Figure 4. Data from 10 tests recorded at 100 kHz is shown in the plot. Excellent repeatability can be observed from the plot. The depth increases to a maximum value and subsequently oscillates, wherein the contact is being unloaded and reloaded without causing further penetration. Hence, the depth up to the point of first maximum represents the elastoplastic regime which is of interest in the current analysis. The maximum depth is reached in less than 500 µs, after which the contact oscillates and hence having fast response and low noise displacement sensors is very important to perform these tests. The load on the sample during this time can be calculated by factoring out the dynamic contribution of the instrument from the force generated by the actuator using Equation (4).

**Figure 5:**
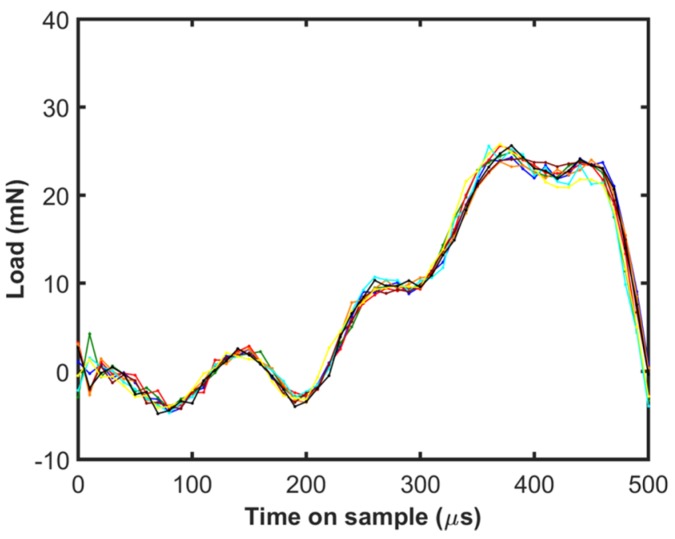
The load on the sample as a function of time on the sample calculated by accounting for the dynamic contribution of the instrument during the step load for 10 different tests shown in different colors.

**Figure 6:**
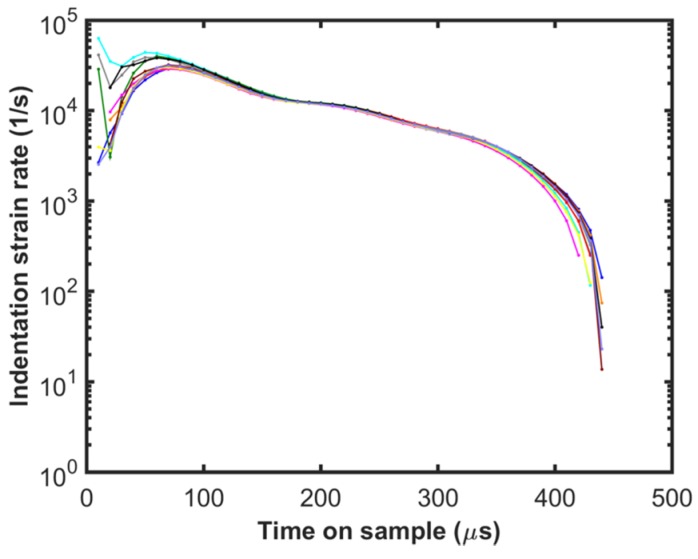
Indentation strain rate as a function of time during the step force input of 16 mN for 10 different tests shown in different colors.

Figure 5 shows the load on sample calculated using Equation (4) for the step load tests. Good repeatability can be observed even in this case, in spite of having to use the first and second derivatives of the displacement signal for load calculations. Similar to the depth-time response shown in Figure 4, the load on sample reaches a maximum value which is at the peak depth. The maximum load on sample (~25 mN) is much higher than the force output of the actuator (16 mN) which is due to dynamic overload from deceleration of the tip. Due to the dynamic nature of this test and the experimental challenges described earlier, analyzing the results of these tests is quite challenging. There are contributions from ISE, the inertia of the instrument, the time constant of the command and measurement signals, and the data acquisition rate which need to be carefully considered. During the initial part of the step load (<100 µs), calculating the acceleration, which is the second derivative of displacement is difficult as there are only 10 data points (data points are 10 µs apart in time). Even though there is good repeatability in the results, data at such short time intervals may not be accurate. As we proceed further in time up to 250 µs, the strain rates are very high (>10^4^ 1/s) as shown in Figure 6, which is beyond the scope of the simple analysis presented in the current work, where a power law behavior is assumed for the strain rate dependence of the flow stress. There may be other physical phenomena that are operative at such high strain rates which are not modeled here. In addition, the ISE is found to exhaust after a depth of 1.2 µm, which corresponds to a time on sample of 340 µs. Furthermore, it is not ideal to use data less than the time constant of the load signal which is 290 µs. In view of the constraints discussed above, step load data beyond 340 µs, up to the point of reaching the maximum depth (440 µs) is useful for the current analysis. It may be noted that the contact unloads completely at ~500 µs and the load on the sample calculation based on Equation (4) is not valid beyond that point.

**Figure 7:**
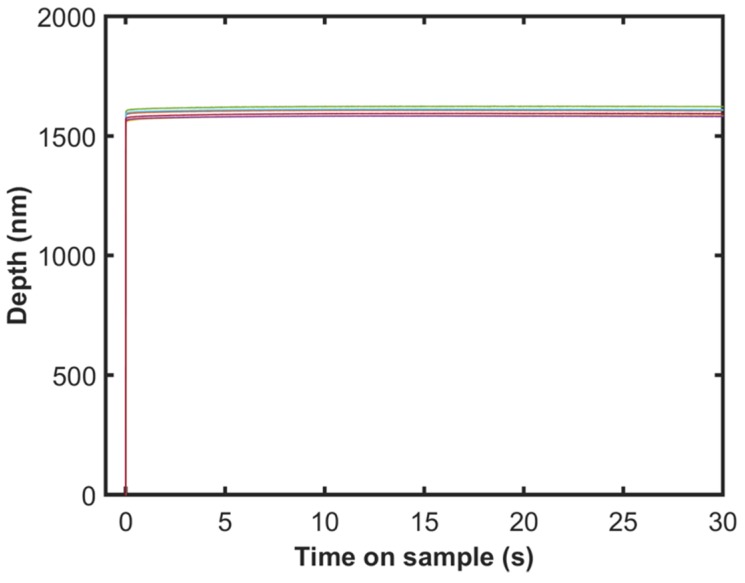
Indentation depth as a function of time during the CLH test for 10 different tests shown in different colors.

Figure 7 shows the indentation depth as a function of time on sample for the CLH tests. The plot shows two distinct regions: the fast initial increase in depth corresponding to the fast load ramp (5 N/s) and the subsequent creep at a fixed force of 16 mN. The creep rate in the hold segment is almost insignificant as expected for aluminum at room temperature and the data may not correspond to the steady state creep as discussed in the previous work of the authors [20].

### 3.2. Strain Rate Effects

**Figure 8:**
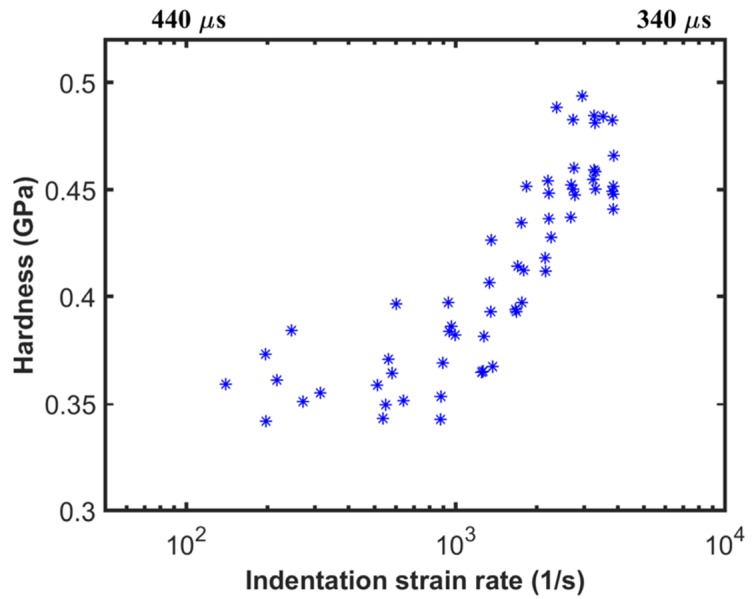
Indentation hardness as a function of indentation strain rate for step load tests.

In this section, we present the strain rate dependence of hardness for the step load tests and the CLH tests. Figure 8 shows the hardness as a function of indentation strain rate for the step load tests. The hardness and the indentation strain rate are calculated using the procedure described in Section 2.4. The plot also shows the time on sample at the extremes of the data. As discussed in Section 3.1, data between 340 µs and 440 µs data is relevant for the current analysis. Within this window, the step load tests enable access to very high strain rates (4000 1/s). In addition, a range of strain rates at the higher end can be accessed in a single test which is simple and quick to perform compared to most conventional high strain rate tests.

**Figure 9:**
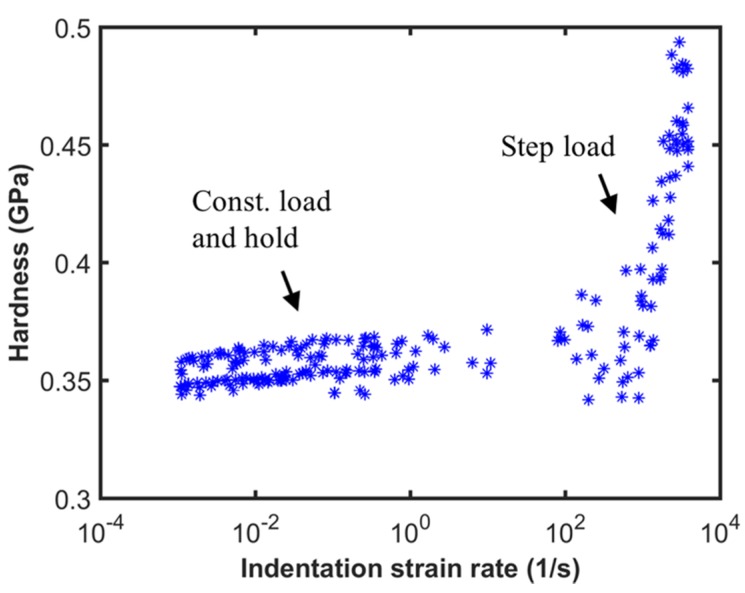
Indentation hardness as a function of strain rate during the constant load and hold (CLH) test and step load test.

Unlike the step load tests, the variation in hardness as a function of indentation strain rate for the CLH tests is calculated from the data in the hold segment. The calculation procedure is similar to the case of step load, but the load on the sample does not have any significant dynamic contribution from the instrument. Figure 9 shows the hardness as a function of indentation strain rate for the CLH tests. The data from the step load tests are also shown for comparison. The data from the CLH tests are above a depth of 1.2 µm where the ISE is not very significant. The plot shows the strength of the current testing methodology wherein a combination of step load tests and CLH tests is used to measure the strain rate dependence of hardness over seven orders of magnitude in the strain rate by a single instrument in a simple and quick manner. It is interesting to note that the strain rate sensitivity, which is the slope of the data, changes around a strain rate of 10^3^ 1/s. This will be discussed in greater detail in the next section.

### 3.3. Equivalent Uniaxial Response

**Figure 10:**
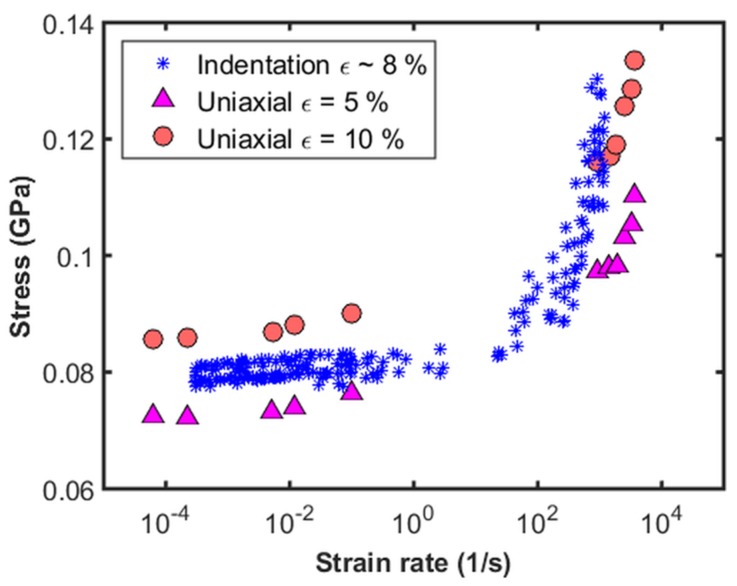
Comparison of equivalent uniaxial strain rate and stress calculated from the indentation data with the uniaxial results from conventional compression test and direct disc impact test (Khan et al. [21]).

In this section, we present the equivalent uniaxial strain rate and stress calculated from the indentation data, and the comparison with the uniaxial data from the literature. The procedure to calculate the equivalent uniaxial strain rate and stress is described in Section 2.5. Figure 10 shows the equivalent uniaxial stress as a function of equivalent uniaxial strain rate calculated from the indentation data and conventional uniaxial testing. Results from uniaxial testing for this alloy over the range of strain rates achieved in this work are obtained from Khan et al. [21]. The low strain rate data is from uniaxial compression testing, while the high strain rate data is from direct disc impact tests. In order to compare the results of this work where a Berkovich tip is used, which induces an equivalent strain of 8%, uniaxial results at two different strains (5% and 10%) are shown in the plot. The indentation data at 8% strain, lies within the region bounded by the uniaxial data at 5% and 10%, over a wide range of strain rates. Given the vast differences in test geometries and test protocols between the uniaxial and indentation tests, it is very interesting to note that the indentation data matches the uniaxial data over seven orders of magnitude in strain rate. The difference in the uniaxial data at two different strain levels indicates that steady state conditions may not have been achieved during indentation which induces 8% equivalent strain. This is similar to the observations of Luthy et al. [22] who performed torsion tests to very high strain (300–1000%) in order to achieve steady state creep in pure aluminum at room temperature. While the results of the current work may not represent steady state conditions, the experimental procedure and analysis presented here provide a generic framework to measure strain rate dependence of stress over a wide range of strain rates with a high degree of precision.

## 4. Summary and Conclusions

Advances in instrumentation have enabled nanomechanical measurements with very low noise levels (sub nanometer) at fast time constants (20 µs) and high data acquisition rates (100 KHz). These capabilities in conjunction with a comprehensive model for instrument’s dynamics and electronics is vital for accurate high strain rate measurements.

The indentation system used in this work is shown to be extremely well characterized by a simple one DOF harmonic oscillator model which can be readily extended to testing under non-ambient conditions (vacuum or high temperature).

A comprehensive model for the instrument’s dynamics and electronics has been presented to accurately factor out the instrument’s contribution to the measurement during a high strain rate test and validated with the step load experiments in free air. The importance of having fast measurement time constants for accurate high strain rate measurements has been demonstrated.

Step load tests have been performed on commercial purity aluminum (1100 Al), wherein the force is ramped to the desired level as fast as the instrument can physically accomplish the change, thereby enabling access to a range of high strain rates (>1000 1/s) in a single indentation test.

A simple procedure has been presented to determine the hardness and indentation strain rate during a step load test. The strain rate dependence of hardness has been measured over seven orders of magnitude by a combination of step load and CLH tests.

The uniaxial equivalent strain rate and stress calculated from the indentation data closely matches the uniaxial results over seven orders of magnitude. Given the vast differences in test geometries and test protocols between the uniaxial and indentation tests, it is very encouraging that the indentation data matches the uniaxial data over several orders of magnitude in strain rate.

The experimental procedure and analysis presented in this work provides a generic framework to measure strain rate dependence of stress over a wide range of strain rates with a high degree of precision in a simple, quick, and cost-effective way compared to the conventional methods. The simplicity of this experimental technique also enables it to be extended to high temperatures, thereby facilitating the measurement of a high strain rate response at elevated temperatures, which is a largely unexplored challenging area of research/experimentation.
